# Effect of 17β-estradiol on a human vaginal *Lactobacillus crispatus* strain

**DOI:** 10.1038/s41598-021-86628-x

**Published:** 2021-03-30

**Authors:** Maximilien Clabaut, Amandine Suet, Pierre-Jean Racine, Ali Tahrioui, Julien Verdon, Magalie Barreau, Olivier Maillot, Agathe Le Tirant, Madina Karsybayeva, Coralie Kremser, Gérard Redziniak, Cécile Duclairoir-Poc, Chantal Pichon, Sylvie Chevalier, Marc G. J. Feuilloley

**Affiliations:** 1 Laboratory of Microbiology Signals and Microenvironment (LMSM EA 4312), Université de Rouen Normandie, 55 rue Saint-Germain, 27000 Evreux, France; 2 Centre de Biophysique Moléculaire, UPR4301 French National Centre for Scientific Research, Orléans, France; 3 Laboratoire EBI, UMR CNRS 7267, Université de Poitiers, Poitiers, France; 4 Seqens Cosmetics’, Porcheville, France; 5 Remedials Laboratory, Paris, France; 6 GymoPharm, Longjumeau, France; 7 Cosmetic Inventions, Antony, France

**Keywords:** Microbiology, Endocrinology

## Abstract

Lactobacilli and estrogens play essential roles in vaginal homeostasis. We investigated the potential direct effect of 17β-estradiol on a vaginal strain of *Lactobacillus crispatus*, the major bacterial species of the vaginal microbiota. 17β-estradiol (10^–6^ to 10^–10^ M) had no effect on *L. crispatus* growth, but markedly affected the membrane dynamics of this bacterium. This effect appeared consistent with a signal transduction process. The surface polarity and aggregation potential of the bacterium were unaffected by exposure to 17β-estradiol, but its mean size was significantly reduced. 17β-estradiol also promoted biosurfactant production by *L. crispatus* and adhesion to vaginal VK2/E6E7 cells, but had little effect on bacterial biofilm formation activity. Bioinformatic analysis of *L. crispatus* identified a membrane lipid raft–associated stomatin/prohibitin/flotillin/HflK domain containing protein as a potential 17β-estradiol binding site. Overall, our results reveal direct effects of 17β-estradiol on *L. crispatus*. These effects are of potential importance in the physiology of the vaginal environment, through the promotion of lactobacillus adhesion to the mucosa and protection against pathogens.

## Introduction

The human microbiota has a dynamic structure that differs among organs and populations^1^. It evolves constantly, in response to local and environmental factors^1–3^. The vaginal microbiota is less diverse than other microbiota^4^ but evolves rapidly with changes in female physiology over time^5^. In young children (under the age of 5 to 6 years), it seems to be composed essentially of microorganisms of cutaneous and fecal origins^6^, but even before menarche, at the age of 10 to 12 years, the vaginal microbiota begins to resemble that of adult women, with a dominance of lactobacilli^7^. Colonization by acid producers^8^ such as *Lactobacillus crispatus, L. iners, L. jensenii* and/or *L. gasseri,* the predominant vaginal bacteria in adults^9, 10^ is associated with decrease in the pH of the vaginal environment. In women of reproductive age, the composition of the vaginal microbiota remains fairly stable until menopause. With a few exceptions, in which lactobacilli remain detectable after menopause^11^, the lactobacilli population generally decreases in size at menopause, resulting in a return of local vaginal pH to neutrality^8^, allowing staphylococci and other skin microorganisms to colonize the vaginal mucosa^11, 12^. Finally, the composition of the post-menopause vaginal microbiota gradually comes to resemble that of the cutaneous microbiota. These events are frequently associated with gynecological problems, including itching, inflammation, and even infections^13^, because lactobacilli naturally provide protection against pathogens. This protection results from the extensive secretory activity of lactobacilli, which produce the lactic acid responsible for the decrease in vaginal pH and key protection factor^14, 15^, but also hydrogen peroxide^8, 16^, exoenzymes, bacteriocins and surfactants^17–19^. For these reasons, various strategies for the re-implantation of lactobacilli in post-menopausal women have been developed, based on the introduction of exogenous bacteria (probiotics), nutritional supplementation (prebiotics) or a combination of both these approaches (symbiotics)^20^. The prevalence of lactobacilli in the vaginal microbiota is of importance for protection against gynecological disorders and sexually transmitted infections^14, 15^, but it remains unclear why the lactobacillus population decreases after menopause. Changes in pH do not seem to be the major factor underlying the decrease in lactobacilli, because, even if the vaginal medium is artificially re-acidified, the lactobacilli population cannot be stabilized and does not exert efficient long-term protection^21^. One reason for the observed changes in this population may be the large decrease in estrogen production during menopause relative to the levels in women of reproductive age, with effects on the dynamics and lubrication of the vaginal mucosa^13, 22^. Low doses of estrogens have been shown to restore a low vaginal pH^23^ and to stimulate glycogen production^24^. However, the potential direct effect of estrogens on lactobacilli has never before been considered.

It has been known since the end of the twentieth century that bacteria can sense and adapt to host factors^25^. The effects of peptide hormones and classical neurotransmitters, in particular, have been studied^26, 27^. However, our knowledge of the impact of steroid hormones remains much more limited. In skin, testosterone indirectly promotes the development of *Cutibacterium acnes* by increasing the availability of surface lipids^28^, and estrogens appear to modulate the composition and metabolic activity of the gut microbiota^29^. Direct effects of estradiol on bacteria have also been reported. In *Agrobacterium tumefaciens* and *Pseudomonas aeruginosa*, estradiol interferes with the expression of virulence by inhibiting inter-bacteria communication^30, 31^. In addition, estradiol stimulates the growth and regulates biofilm formation, coaggregation and polysaccharide production in *Prevotella intermedia*^32^. An effect of estradiol on *Staphylococcus epidermidis* growth and biofilm formation has also been reported^33^. However, the effects of estradiol on lactobacilli remain unknown.

In this study, we investigated the effects of estradiol on a vaginal strain of *L. crispatus*, the genome of which was recently sequenced^34^, through cell-binding assays and physiological, physicochemical, morphological, and bioinformatics approaches. We found that estradiol can affect the physiology of lactobacilli, suggesting a possible key role for estradiol in their adherence to the vaginal mucosa.

## Results

### 17β-estradiol alters *L. crispatus* CIP104459 membrane fluidity

**Figure 1:**
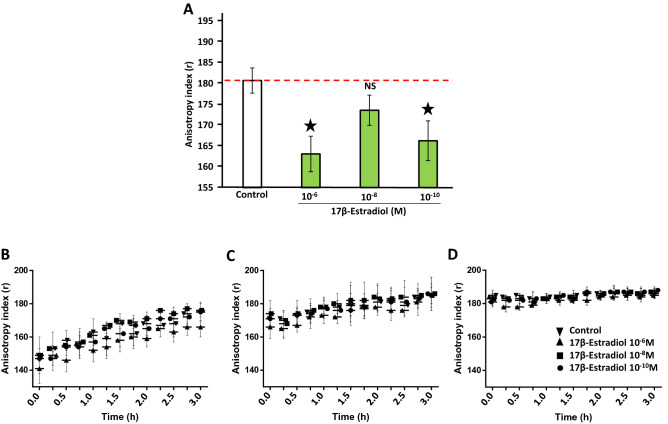
Effect of 17β-estradiol on *Lactobacillus crispatus* CIP104459 membrane fluidity. (**A**) Membrane fluidity (anisotropy index) of *L. crispatus* CIP104459 grown for 18 h in the absence (control) or presence of 17β-estradiol (10^–6^, 10^–8^ and 10^–10^ M). (**B**) Membrane fluidity (anisotropy index) of *L. crispatus* grown for 6 h in control MRS medium and then exposed to 17β-estradiol (10^–6^, 10^–8^ or 10^–10^ M). (**C**) Membrane fluidity (anisotropy index) of *L. crispatus* grown for 12 h in control MRS and subsequently exposed to 17β-estradiol (10^–6^, 10^–8^ or 10^–10^ M). (**D**) Membrane fluidity (anisotropy index) of *L. crispatus* grown for 24 h in control MRS medium and subsequently exposed to 17β-estradiol (10^–6^, 10^–8^ and 10^–10^ M). Values and curves are the means ± SEM of three independent studies. (NS: not significant; * = *p* < 0.05).

Preliminary studies showed that 17β-estradiol, at concentrations of 10^–6^, 10^–8^ and 10^–10^ M, had no effect on *L. crispatus* CIP104459 growth (Suppl Fig. 1). 17β-estradiol is amphiphilic and can, therefore, integrate into phospholipid membranes, affecting their fluidity. We investigated the effect of 17β-estradiol on *L. crispatus* CIP104459 membrane fluidity, by performing fluorescence anisotropy analysis, as previously described^35^. Anisotropy reflects the degree of organization of the bacterial membrane, with decreases in this index indicating a less organized phospholipid bilayer and, thus, an increase in membrane fluidity. Bacteria were grown for 18 h in the presence or absence of 17β-estradiol at concentrations of 10^–6^, 10^–8^ and 10^–10^ M, and anisotropy index was then measured. 17β-estradiol decreased fluorescence anisotropy (Fig. 1A). This effect was statistically significant for 17β-estradiol concentrations of 10^–6^ and 10^–10^ M. For 10^–8^ M, the difference relative to control values was smaller and non-significant. We initially suspected that this result was an artefact, but subsequent studies showed that the bacterial response to 17β-estradiol was not systematically linear, suggesting a complex mechanism and different targets. These data indicate that 17β-estradiol increases membrane fluidity in *L. crispatus* CIP104459. This increased fluidity may be due to the integration of 17β-estradiol into the bacterial membrane or an adaptive response of the bacteria after the sensing of 17β-estradiol as an environmental signal. The sensitivity of the bacterial membrane changes with the growth stage^36^ and, if it happens, 17β-estradiol is rapidly integrated into the membrane, within minutes or hours^37^. The bacteria were then cultured for 6, 12 or 24 h (corresponding to mid-log growth phase, early stationary growth phase and full stationary growth phase, respectively) in MRS medium before the addition of 17β-estradiol, and anisotropy was immediately monitored at 15-min intervals for 3 h (Fig. 1B–D, respectively). Remarkably, the fluorescence anisotropy of control bacteria after 6 h of culture, and, to a lesser extent, after 12 h, tended to increase spontaneously. No variation was detected when bacteria were grown to stationary phase (24 h), suggesting that a steady state membrane organization had been achieved by this point. The exposure of bacteria to 17β-estradiol after 6 h of culture resulted in noisy anisotropy data, probably because of membrane instability, but a gradual evolution of the signal was observed over 3 h, as for the control. The signal was more stable for bacteria cultured for 12 and 24 h before treatment with 17β-estradiol, for which no difference relative to the control was noted. If the effect of 17β-estradiol was due to non-specific interaction with the membrane, this process should be rapid and associated with an immediate decrease in anisotropy. Limited changes in membrane fluidity were observed for cultures treated after 6 h, whereas such changes were undetectable in cells grown to stationary phase. These data thus indicate that the increase in *L. crispatus* CIP104459 membrane fluidity observed after exposure to 17β-estradiol from the beginning to the end of culture, probably results from an adaptative process. These exposure conditions were used in subsequent assays.

### 17β-Estradiol marginally affects the polarity and Lewis acid/base surface properties of *L. crispatus* CIP104459

**Figure 2:**
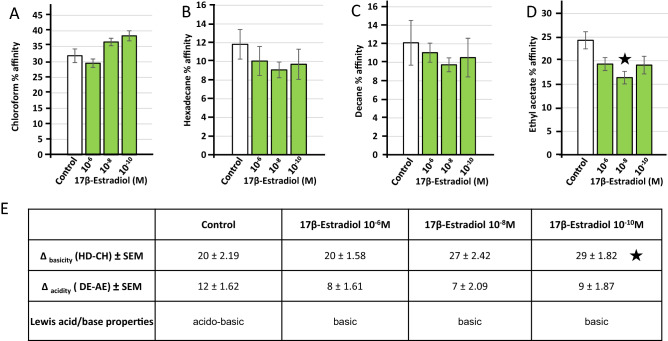
Effect of 17β-estradiol (10^–6^, 10^–8^ and 10^–10^ M) on the solvent affinities and Lewis acid/base surface properties of *Lactobacillus crispatus* CIP104459. (**A**) Partitioning between water and chloroform. (**B**) Partitioning between water and hexadecane. (**C**) Partitioning between water and decane. (**D**) Partitioning between water and ethyl acetate. (**E**) Lewis acid/base behavior of *L. crispatus* CIP104459 with the two solvent couples hexadecane (HD)/chloroform (CH) and decane (DE)/ethyl acetate (EA). The values presented are the means ± SEM of three independent studies. (* = *p* < 0.05).

Given the effect of 17β-estradiol on membrane fluidity, we assessed the surface polarity of *L. crispatus* CIP104459 with the microbial affinity to solvents (MATS) assay. Solvent affinity was limited for this bacterium, particularly for apolar solvents, such as decane and hexadecane (mean values 12 ± 2.19% and 12 ± 1.36%, respectively). Affinity for more polar solvents, such as ethyl acetate and chloroform, was higher (24 ± 1.21% and 32 ± 1.62%, respectively) (Fig. 2A–D). *L. crispatus* CIP104459 thus has a hydrophilic surface. Exposure to 17β-estradiol tended to lead to a limited increase in affinity for chloroform and a parallel decrease in affinity for the other three solvents. Only the decrease in affinity for ethyl acetate with 17β-estradiol at 10^–8^ M was statistically significant (*p* < 0.05). An evaluation of the Lewis acid/base ratio with two pairs of solvents, hexadecane/chloroform and decane/ethyl acetate, revealed that all bacteria exposed to 17β-estradiol displayed a change in their electron donor/acceptor behavior. The untreated bacterial surface had acido-basic characteristics, whereas the surface of 17β-estradiol treated bacteria was basic (Fig. 2E). However, this change was marginal and was significant only for hexadecane/chloroform, for bacteria exposed to 10^–10^ M 17β-estradiol.

### 17β-Estradiol alters the morphology of *L. crispatus* CIP104459, but not its aggregation phenotype

**Figure 3:**
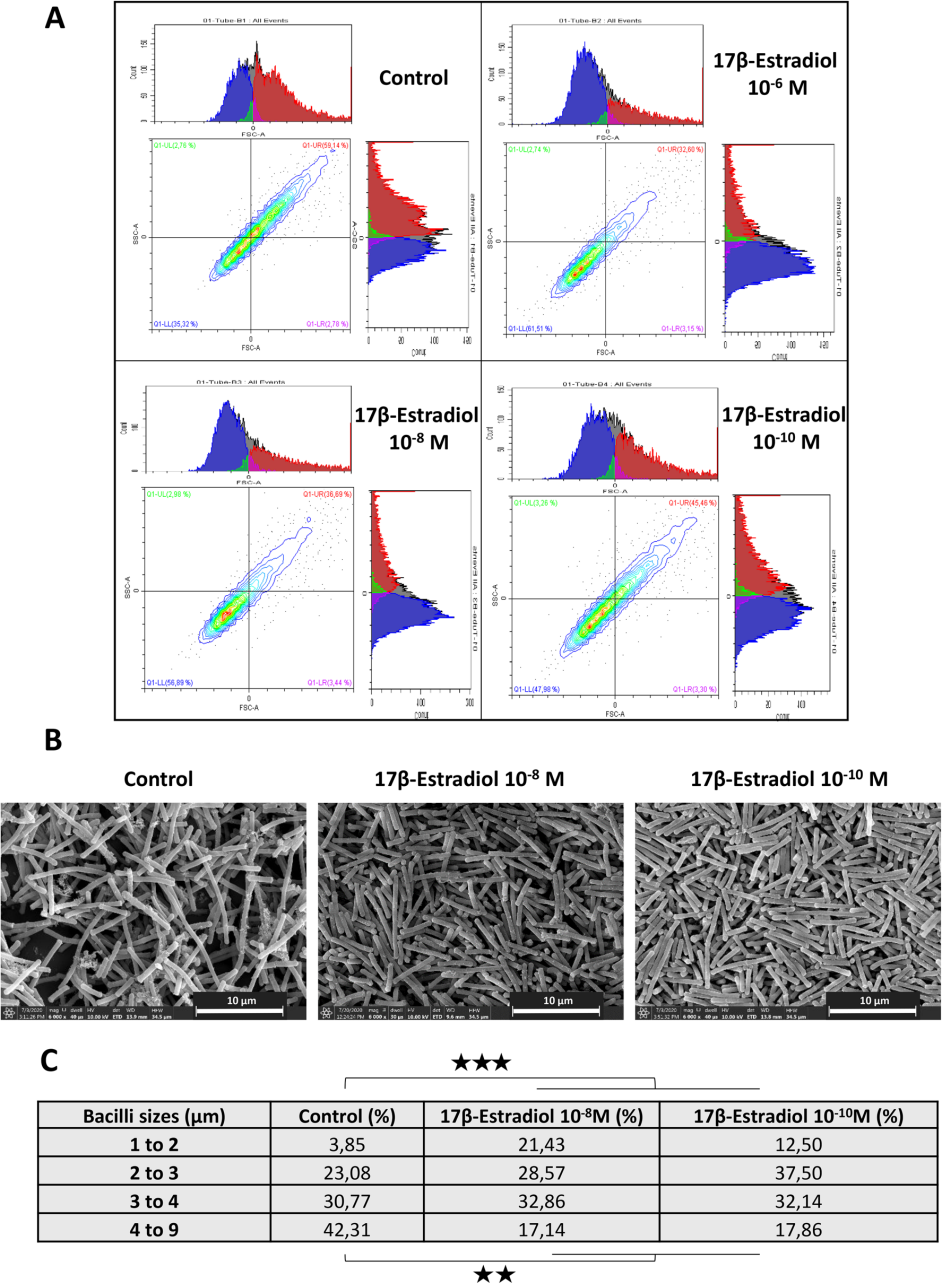
Effect of 17β-estradiol on the aggregation potential and morphology of *Lactobacillus crispatus* CIP104459. (**A**) Flow cytometry diagrams illustrating the effect of 17β-estradiol (10^–6^, 10^–8^ and 10^–10^ M) on the mean size and aggregation (*x* axis = forward scattering fraction (FSC)) and surface heterogeneity (*y* axis = side scatter (SSC)) of the bacteria. (**B**) Scanning electron microscopy of control and 17β-estradiol treated bacteria. Scale bar = 5 µm. (**C**) Table showing the relative percentages of bacteria classified by size after culture in the presence or absence of 17β-estradiol (10^–8^ or 10^–10^ M). (** = *p* < 0.01; *** = *p* < 0.001).

As membrane fluidity can affect cell morphology or aggregation, we then investigated the effects of 17β-estradiol on these phenotypes in *L. crispatus* CIP104459. For macroscopic investigations of the aggregation phenotype, we first performed a “sedimentation” technique, as previously described by Vandevoorde et al.^38^. 17β-estradiol had no effect on the aggregation potential of *L. crispatus* CIP104459 in these conditions. The percentage aggregation reached a mean value of 66.6 ± 1.6% in control bacteria and of 65.3 ± 2.2, 62.9 ± 1.3, and 66.4 ± 1.2% in bacteria cultured in the presence of 10^–6^, 10^–8^ and 10^–10^ M 17β-estradiol, respectively (Suppl. Fig. 2). The effect of 17β-estradiol on *L. crispatus* CIP104459 was assessed further by flow cytometry, which can provide information about cell aggregation, mean size, and structural heterogeneity (granularity). This information is obtained by determining the forward scatter (FSC) and lateral or side scatter (SSC) fractions of the light^39^. We plotted the FSC and SSC values of *L. crispatus* CIP104459. For FSC (*x* axis), the maximal size of the detected events (in red) was decreased by exposure to 17β-estradiol, whereas the signal corresponding to small events (in blue) increased (Fig. 3A). This pattern was particularly marked for bacteria exposed to 10^–6^ and 10^–8^ M 17β-estradiol, and was less clear for those exposed to 10^–10^ M of 17β-estradiol, although small particles continued to predominate, relative to control conditions. By contrast, the surface heterogeneity (granularity) of the bacteria, corresponding to the SCC values (*y* axis), was not modified. The difference in FSC values between control and 17β-estradiol-treated bacteria was not significant, probably because this signal combines two different types of information, relating to the degree of aggregation of the bacteria and their mean size. Overall, these data suggest that 17β-estradiol affects the morphology of *L. crispatus* CIP104459. Scanning electron microscopy (SEM) observations of cells revealed that 17β-estradiol had no detectable effect on aggregate formation (Fig. 3B). Measurements of cells’ length on SEM images led to *L. crispatus* CIP104459 cells being classified as small (1 to 2 µm) to large (4 to 9 µm). Remarkably, treatment with 10^–8^ or 10^–10^ M 17β-estradiol led to significant increases in the proportion of small bacteria (*p* < 0.001) and decreases in the portion of large bacteria (*p* < 0.01) (Fig. 3C).

### 17β-Estradiol promotes biosurfactant production by *L. crispatus* CIP104459

**Figure 4:**
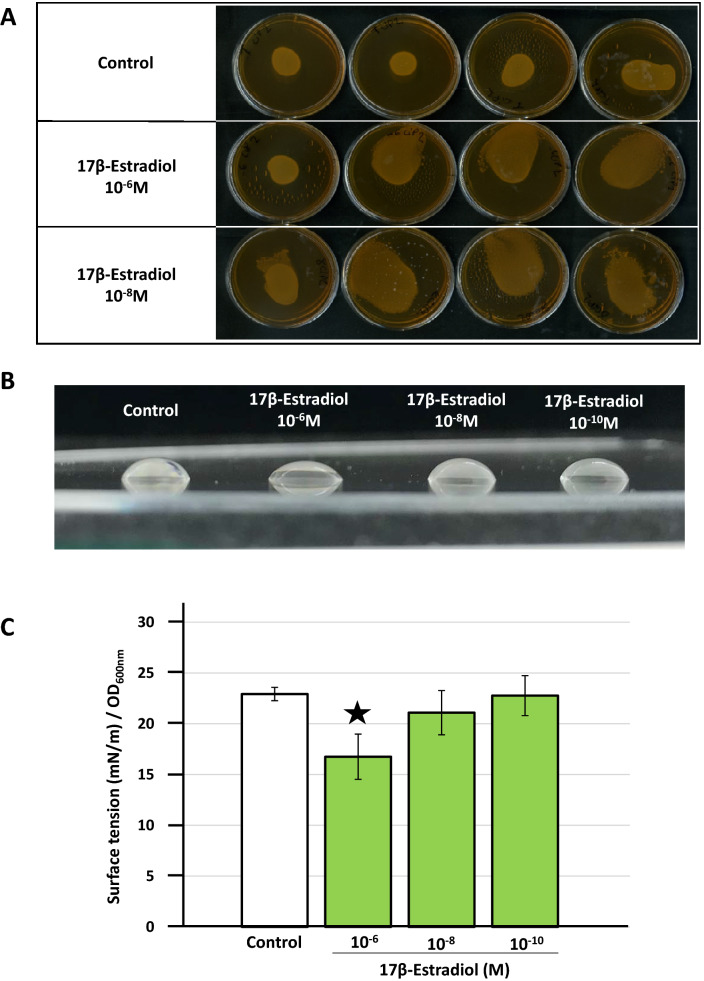
Effect of 17β-estradiol on *Lactobacillus crispatus* CIP104459 biosurfactant production. (**A**) Cultures of *L. crispatus* CIP104459 on MRS agar medium after exposure to 17β-estradiol (10^–6^ and 10^–8^ M). (**B**) Typical shape of drops of solution obtained after rinsing the bacterial lawn formed on Petri dishes in the presence or absence of 17β-estradiol (10^–6^, 10^–8^ and 10^–10^ M). (**C**) Surface tension value of solutions extracted from *L. crispatus* CIP104459 exposed to 10^–6^, 10^–8^ and 10^–10^ M 17β-estradiol, measured by the pendant drop method. Values are the means ± SEM of three independent experiments. (* = *p* < 0.05).

Biosurfactants are surface-active molecules that can reduce air/water surface tension to low levels. Some of these molecules can insert into bacterial membranes and modify membrane fluidity^40^. Cultures of *L. crispatus* CIP104459 exposed to 17β-estradiol (10^–6^ and 10^–8^ M) for 48 h and layered on MRS-agar spread and flowed over the Petri dishes, suggesting that biosurfactants were produced (Fig. 4A**)**. Biosurfactants were extracted by gently scrapping the bacterial lawn and resuspending the cells in Volvic water (selected for its neutral effect on surface tension). The bacteria were then removed by centrifugation, as described by Meylheuc et al*.*^41^. The solutions obtained from *L. crispatus* cultures with and without exposure to 17β-estradiol (10^–6^, 10^–8^ and 10^–10^ M) behaved differently on testing by the sessile drop technique on a polystyrene surface (Fig. 4B). Controls, with 10^–6^ to 10^–10^ M 17β-estradiol alone in Volvic water, showed that the steroid alone had no effect on the surface tension values (*data not shown*). The drops of solution obtained from the bacterial lawn exposed to 17β-estradiol were flatter than those obtained with pure Volvic water (control), particularly for samples treated with 10^–6^ M 17β-estradiol. This decrease in the contact angle of the liquid with the surface indicates a decrease in surface tension due to the presence of biosurfactants. We used the pendant drop method to determine the decrease in surface tension, and the mean surface tension of the solution was calculated by modeling the shape of the hanging drop visualized with a camera. A dose-related decrease in the surface tension of solutions extracted from *L. crispatus* CIP104459 exposed to 10^–6^, 10^–8^ and 10^–10^ M 17β-estradiol was observed. However, the difference in surface tension between control solutions and solutions from 17β-estradiol-treated bacteria was significant only for 10^–6^ M 17β-estradiol (Fig. 4C).

### 17β-Estradiol increases the adhesion of *L. crispatus* CIP104459 to vaginal mucosa epithelial cells in vitro, but has a limited effect on biofilm formation

**Figure 5:**
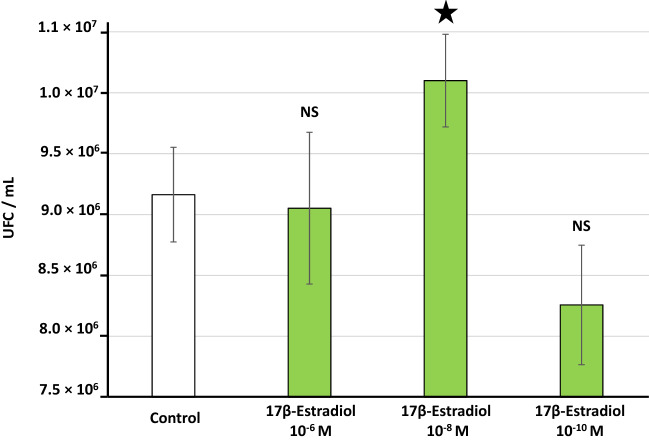
Effect of 17β-estradiol on the adhesion potential to VK2/E6E7 human vaginal cells of *Lactobacillus crispatus* CIP104459 grown in De Man, Rogosa and Sharpe (MRS) medium. Values are the means ± SEM of three independent studies. (*NS* not significant; * = *p* < 0.05).

**Figure 6:**
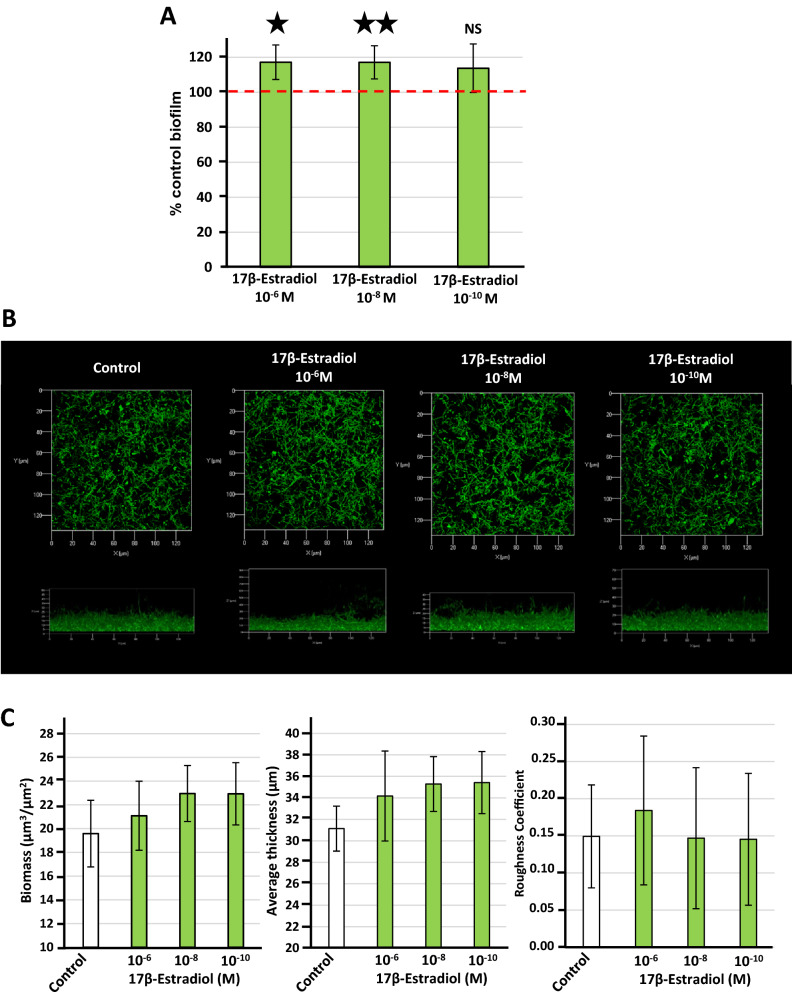
Effect of 17β-estradiol (10^–6^, 10^–8^ and 10^–10^ M) on the biofilm formation activity of *Lactobacillus crispatus* CIP104459. (**A**) Biofilm formation of bacteria grown in De Man, Rogosa and Sharpe (MRS) medium in the presence or absence of 17β-estradiol studied by the crystal violet technique. The dotted line indicates the control level (100%). (**B**) Structure of *Lactobacillus crispatus* CIP104459 biofilm formed in simulating genital tract secretion (SGTS) medium and visualized by confocal laser scanning microscopy. All figures are represented at the same scale and show top views (x/y) and lateral views (x/z mean thickness) of the biofilms formed in the presence or absence of 17β-estradiol. (**C**) Effect of 17β-estradiol on the calculated means biofilm biomass, thickness and roughness. Results are representative of three independent experiments. (*NS* not significant; * = *p* < 0.05; ** = *p* < 0.01).

Finally, we investigated the effect of 17β-estradiol on adhesion and biofilm formation. We assessed the adhesion of *L. crispatus* CIP104459 to vaginal mucosa epithelial cells with the human vaginal VK2/E6E7 cell line. Bacteria cultured in the presence of 10^–8^ M 17β-estradiol displayed significantly higher rates of adhesion to VK2/E6E7 cells (+ 10.2 ± 3.8%) (Fig. 5). Unexpectedly, this effect was not observed with bacteria exposed to 10^–6^ M and 10^–10^ M 17β-estradiol. Assessment of *L. crispatus* CIP104459 biofilm formation in crystal violet staining assays showed that 10^–6^ and 10^–8^ M 17β-estradiol induced a small but significant increase in biofilm formation, whereas 10^–10^ M 17β-estradiol did not (Fig. 6A). We investigated the effect of 17β-estradiol on biofilm architecture in more details, by visualizing the 3D structure of *L. crispatus* CIP104459 biofilm by confocal laser scanning microscopy. However, bacteria cultured in MRS were unable to adhere to the glass bottom of the microtiter plate, and no biofilm was observed in such conditions (*data not shown*). We therefore cultured the bacteria in a specific medium, “simulating genital tract secretion” (SGTS) medium^42^ (Table 1). In these conditions, 17β-estradiol had no visible effect on biofilm structure (Fig. 6B). Biofilm images analysis with COMSTAT2 software revealed that the mean thickness (μm), mean biomass volume (μm^3^/μm^2^) and roughness coefficients of control and 17β-estradiol-treated bacteria were unaffected by treatment (Fig. 6C).

**Table 1:** Composition of the simulating genital tract secretion (SGTS) medium.

Component	Final concentration (g/L)
**Part 1**^a^	
NaCl	3.5
KCl	1.5
K_2_HPO_4_	1.74
KH_2_PO_4_	1.36
Dextrose	10.8
Cysteine HCl	0.5
**Part 2**^b^	
Glycogen	1
Mucin	0.25
Tween 20	0.2
Urea	0.5
Hemin	0.5
Albumine	0.2
MgSO_4_*	0.3
NaHCO_3_*	0.04
**Part 3**	
Vitamin mix^d^	5 mL of 100X solution

*Solutions were sterilized by passage through a membrane filter (pore size, 0.22 µm).

^a^Prepared by dissolving the components in pure 18.2 MΩ water. After adjustment of the pH to 7.2 with NaOH, the solution was autoclaved for 15 min at 121 °C and then cooled to room temperature.

^b^Part 2 consisted of eight components prepared separately. Components 1 to 5 were prepared as concentrated solutions and autoclaved for 15 min at 121 °C.

### Bioinformatic study of the potential *L. crispatus* CIP104459 17β-estradiol receptor

**Figure 7:**
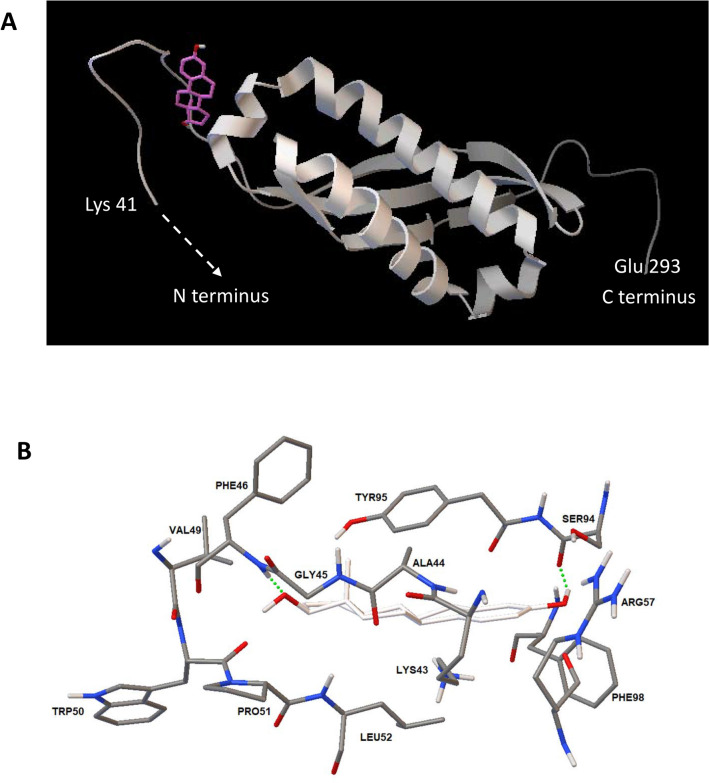
3D modeling of the potential interaction of the *Lactobacillus crispatus* CIP104459 SPFH domain-containing protein (NCBI Reference Sequence: WP_013086692.1) with 17β-estradiol. (**A**) Illustration of the sub-membrane domain of the *L. crispatus* SPFH domain-containing protein showing its potential interaction with 17β-estradiol (pink). (**B**) Calculated 17β-estradiol binding site on the *L. crispatus* SPFH domain-containing protein determined with AutoDock 4.2 (17β-estradiol appears in white and the hydrogen bonds between 17β-estradiol and amino acids are shown as green dotted lines).

As shown here, 17β-estradiol affects on the physiology of *L. crispatus* CIP104459 at concentrations as low as 10^–8^ or 10^–10^ M for some phenotypes, suggesting the existence of a putative sensor for estradiol in this bacterium. The potential binding site for 17β-estradiol was investigated by a bioinformatic approach based on the draft genome of *L. crispatus* CIP104459^34^, which was compared with the recently annotated genome of *L. crispatus* CO3MRSI1^43^. The putative translated ORFs were compared to the published sequences of nine eukaryotic 17β-estradiol binding proteins identified in previous studies (Table 2). Two of these proteins, estrogen-related receptor gamma (ERR3, UniProtKB—P62508) and the prohibitin-2 (PHB2, UniProtKB—Q99623) displayed significant identity (33 and 24%, respectively) and similarity (55 and 46%, respectively) to a *L. crispatus* CIP104459 protein, the SPFH domain-containing protein (NCBI Reference Sequence: WP_013086692.1). Based on the structure of the binding site in eukaryotic proteins, the potential association between 17β-estradiol and the *L. crispatus* SPFH domain-containing protein was studied with AutoDock 4.2^44^. This 293 amino acids protein contains two domains, an N-terminal domain between amino acids 1 and 40 corresponding to a short extracellular sequence (amino acids 1 to 9), and a transmembrane helix (amino acids 10 to 25) with a long C-terminal submembrane domain organized as an α helix and a β-sheet, respectively. A potential binding site for 17β-estradiol was identified in the submembrane domain, in a region before the α-helix and β-sheet folds (Fig. 7A). The principal amino acids involved in interactions between 17β-estradiol and the SPFH protein appear to be a glycine 45 and serine 94 (Fig. 7B). The binding value obtained (− 7.8 kcal/mol) was particularly high and was significant, as it was identical in 53% of the calculated clusters.

**Table 2:** List and references of the nine eukaryotic 17β-estradiol binding proteins tested for potential homology to *Lactobacillus crispatus* CIP104459 proteins.

UniProtKB accession number	Human gene	Name
P03372	ESR1	ESR1_HUMAN Estrogen receptor
O95718	ESRRB	ERR2_HUMAN Steroid hormone receptor ERR2
Q99527	GPER1	GPER1_HUMAN G-protein-coupled estrogen receptor 1
P62508	ESRRG	ERR3_HUMAN Estrogen-related receptor gamma
Q92731	ESR2	ESR2_HUMAN Estrogen receptor beta
Q99623	PHB2	PHB2_HUMAN Prohibitin-2
Q8NI08	NCOA7	NCOA7_HUMAN Nuclear receptor coactivator 7
P49888	SULT1E1	ST1E1_HUMAN Estrogen sulfotransferase
Q86YN6	PPARGC1B	PRGC2_HUMAN Peroxisome proliferator-activated receptor gamma coactivator 1-beta

## Discussion

There is now ample evidence that the response of human commensal bacteria to peptide hormones and neurohormones is essential for skin homeostasis^45^. Cutaneous bacteria, such as *C. acnes,* have been shown to express sensors for catecholamines (norepinephrine and epinephrine), suggesting that *C. acnes* acts as a relay between stress and acne^46^. Bacteria even produce small eukaryotic communication molecules, such as histamine, glutamine and, γ-aminobutyric acid (GABA) in particular^45^. GABA is even considered to be the universal inter-kingdom communication factor^47^. Conversely, sterols are rare in bacteria^48^, and the influence of steroids on bacterial physiology remains poorly documented, despite the development of bacteria in environments in which steroid hormones have a crucial impact.

The absence of an effect of 17β-estradiol on the growth of *L. crispatus* CIP104459 is consistent with previous studies showing that human neurohormones and neurotransmitters, including substance P, calcitonin gene related peptide (CGRP), natriuretic peptides, GABA and catecholamines, generally have no effect on the cultivability of commensal bacteria^45^. Conversely, steroid hormones, such as estradiol and androstenedione, inhibit the growth of *Helicobacter pylori*^49^. In this species, progesterone and other cholesterol derivatives may even have lethal effects, which have been attributed to membrane damage^49, 50^. However, these effects were observed only at high concentrations (> 50 µM)^49^ and, at these non-physiological doses, many eukaryotic hormones, including the aforementioned peptides, can also have non-specific anti-microbial activities^26^. Moreover, this antimicrobial activity of steroids is species dependent, as sterols have been shown to have limited effects on viability of the Gram-negative spirochete *Borrelia burgdorferi*, with effects membrane permeability^51^. *Helicobacter* and *Borrelia* are representative of a small group of bacteria with large amounts of cholesterol in their membranes, whereas *Lactobacillus crispatus* is a Gram-positive Firmicute with only small amounts of sterols in its membrane^48^.

We assessed the effect of 17β-estradiol on *L. crispatus* membrane fluidity by measuring membrane anisotropy. A decrease in membrane anisotropy was observed after continuous exposure of the bacteria to 17β-estradiol, indicating greater fluidity^35^, consistent with changes in membrane homoeostasis. Many bacteria use the sterol-like molecules hopanoids to regulate their membrane fluidity in response to changes in environmental conditions, such as temperature shifts^52, 53^, which suggests that 17β-estradiol may be able to insert into *L. crispatus* membranes. We investigated this mechanism further by analyzing membrane fluidity following short-term exposure to 17β-estradiol. Minor differences in fluorescence anisotropy index were observed in bacteria grown to mid-exponential growth phase (6 h of growth). Remarkably, this phenomenon was attenuated in bacteria taken to the start of stationary phase before exposure to 17β-estradiol (12 h of growth), and disappeared completely in bacteria in late stationary phase exposed to 17β-estradiol. Our data therefore suggest that the increase in membrane fluidity resulting from continuous bacterial exposure to 17β-estradiol may be due an adaptive response of the bacteria involving hormone signal transduction, rather than steroid insertion into the membrane. This hypothesis is also supported by the conservation of the response to 17β-estradiol at a concentration of 10^–10^ M, a concentration at which the number of steroid molecules accessible per bacterium should be very small, and insufficient for direct physico-chemical effects.

We performed MATS assay to investigate the surface polarity of *L. crispatus* exposed to 17β-estradiol. The changes observed were small, and were significant for only one solvent (ethyl acetate) and for an estradiol concentration of 10^–8^ M. In parallel, the Lewis acid/base properties of the bacterial surface shifted from acido-basic to basic, indicating that 17β-estradiol treatment had little effect on cell surface polarity. In addition, flow cytometry studies showed that the surface heterogeneity of the bacteria exposed to 17β-estradiol remained similar to that observed in control conditions. Conversely, the mean size of the particles detected by flow cytometry tended to decrease after exposure. All lactobacilli can aggregate and *L. crispatus* is one of the species in which this potential is greatest^54^. However, SEM observations of *L. crispatus* revealed that 17β-estradiol induced a significant decrease in the mean size of bacteria, suggesting that the smaller particle observed on flow cytometry were due to the presence of smaller *L. crispatus* cells rather than a difference in aggregation potential. Consistent with this hypothesis, the aggregation potential of lactobacilli has been shown to depend on outer surface S-layer proteins^55^, and we found here that the physico-chemical properties of the bacterial surface were little affected by 17β-estradiol exposure. Nonetheless, this study reveals that the response of *L. crispatus* to 17β-estradiol involves a complex process leading to changes in the bacterial size.

*L. crispatus* CIP104459 colonies exposed to 17β-estradiol have flattened, leaky phenotypes on Petri dishes. These observations suggested an increase in biosurfactant production. Biosurfactants are surface-active molecules that can decrease air/water surface tension to low levels, but can also insert into biotic or abiotic interfaces. Some biosurfactants can insert into bacterial membranes and modify membrane fluidity^40^. The observed decrease in surface tension was dose related but significant only in the presence of 10^–6^ M estradiol. Many lactobacilli are known to produce molecules with biosurfactant activity, including glycoproteins, such as surlactin^56^. Vaginal *L. crispatus* has recently been shown to produce biosurfactant^57^. The molecule concerned appears to be a complex non-homogeneous lipopeptide, because sophisticated chemical techniques, including Fourier-transformed infrared spectroscopy and electron spray mass spectrometry were unable to determine its complete structure^57^. This *L. crispatus* biosurfactant has interesting properties, including low cytotoxicity, the stimulation of mucoadhesion and antagonism to the adhesion of *Candida *spp^57^. The effect of 17β-estradiol on *L. crispatus* biosurfactant production is of interest because this species is one of the major species of the vaginal microbiota^4, 5, 10, 12^, with potential direct consequences for the preservation of vaginal homeostasis. This hypothesis is supported by the observation that 10^–8^ M 17β-estradiol increased the adhesion of *L. crispatus* CIP104459 to VK2/E6E7 vaginal cells. Unexpectedly, higher or lower concentrations of 17β-estradiol had no significant effect. However, this may be accounted for by differences in biosurfactant behavior at different concentrations. Indeed, the surfactant behavior related to their amphiphilic character varies with concentrations, up to a specific limit, known as the critical micellar concentration, but biosurfactants can saturate any interface, and micelle formation modifies interactions with the environment^58^. Biosurfactants must, thus, be present at an optimal concentration to exert their activity, and a concentration of 10^–8^ M should be in the range within which 17β-estradiol stimulates *L. crispatus* biosurfactant production optimally.

Biofilm formation also depends on bacterial adhesive properties, and other parameters, including the secretory activity and matrix composition, may also play essential roles. Here, crystal violet staining assays showed that 17β-estradiol induced a limited, but generally significant, increase in biofilm formation. Crystal violet is not specific and labels both bacterial bodies and the matrix. We then characterized the effect of 17β-estradiol more precisely, by studying the structure of the *L. crispatus* biofilm by confocal microscopy. No difference in biofilm structure or mean thickness was observed. For crystal violet assays, the bacteria were grown in MRS medium and biofilm formation was studied in polystyrene tubes. By contrast, for confocal microscopy, bacteria had to be grown in another medium (SGTS), and the biofilm was formed on a glass surface. These technical differences may account for the absence of an effect of 17β-estradiol on biofilm formation by confocal microscopy. Nevertheless, estradiol appears to have a marginal impact on biofilm formation.

We used a bioinformatic approach based on the published draft genome of *L. crispatus* CIP104459^34^ to identify the potential 17β-estradiol binding protein. For reference, we selected a series of nine molecules identified as estradiol-binding proteins in eukaryotes. Two of these proteins, estrogen-related receptor gamma (ERR3) and prohibitin-2 (PHB2), displayed significant similarity and identity to a *L. crispatus* CIP104459 protein, the SPFH domain-containing protein. SPFH stand for “stomatin, prohibitin, flotillin and HflK/C” and proteins of the SPFH family are associated with membrane lipid rafts^59^. The values obtained for binding between 17β-estradiol and the SPFH domain containing protein were particularly high (− 7.8 kcal/mol), indicating a strong probability of interaction. The presence of the 17β-estradiol binding site in the submembrane region of the SPFH protein is consistent with the ability of 17β-estradiol to cross membranes freely, due to its amphiphilic structure. Experimental studies in the Gram-negative bacterium *B. burgdorferi* also suggested an association of sterols with lipid rafts^48^. Moreover, it has been shown that these membrane microdomains should have functions that are more than simply structural in bacteria, with at least one of these functions based on signal transduction^60^. Collectively, our data therefore suggest a putative association of 17β-estradiol with the SPFH domain containing protein in *L. crispatus* membrane lipid rafts, serving as the first step in the induction of the bacterial response.

Taken together, our findings reveal that 17β-estradiol can exert direct effects on *L. crispatus* by modifying its morphology and inducing biosurfactant production. This process may be important in the physiology of the vaginal environment, promoting the adhesion of *L. crispatus* to the mucosa. It is also consistent with the mean normal concentration of estradiol in vaginal fluid (135 pg/mL, i.e. 5 × 10^–7^ M/L)^61^. The antimicrobial activity of *L. crispatus* and the protection it provides appears to be strain dependent, as shown in a previous study^62^, and these results require confirmation in unrelated strains, and even other *Lactobacillus* species. Our results suggest that it may be possible to act on lactobacilli through estrogen or estrogen-like compounds as part of a potential new strategy for preserving or restoring vaginal homeostasis.

## Methods

### Bacterial strain and culture conditions

*Lactobacillus crispatus* CIP104459 was obtained from the Institut Pasteur Collection (CRBIP-Microorganism biobank catalogue, Paris, France). It was isolated in 1955 from a vaginal swab from a non-menopausal woman at the “La Croix-Rousse” maternity center (Lyon, France). In our laboratory, this strain was stored at − 140 °C in a cryofreezer (Thermo Fisher Scientific, Waltham, Massachusetts, USA). The draft genome sequence of *L. crispatus* CIP 104459 has recently been determined and deposited in the DDBJ/ENA/GenBank under accession number VOMA00000000^34^. This bacterium was grown in anoxic conditions in conical 15 mL tubes (Corning, Thermo Fisher Scientific, Waltham, Massachusetts, USA) containing (filled to maximal capacity*)* De Man, Rogosa and Sharpe (MRS) medium (VWR, Fontenay-sous-Bois, France), at 37 °C, without shaking. Stock cultures in glycerol 30% (v/v) MRS were prepared and stored at − 80 °C before use. Precultures in stationary phase (OD_600 nm_ = 1.1 ± 0.1) were prepared in MRS in anoxic conditions, at 37 °C for 48 h. The density of the bacterial suspensions was determined by measuring optical density at 600 nm with a spectrophotometer (ThermoSpectronics, Cambridge, UK). The absence of contamination was checked by plating onto MRS agar in Petri dishes (VWR, Fontenay-sous-Bois, France). The growth of *L. crispatus* CIP104459 was monitored over a period of 48 h.

For the monitoring of growth kinetics, bacteria were layered in microplates, at an initial OD_600 nm_ of 0.08 and incubated in anoxic conditions for 48 h at 37 °C with shaking (360 rpm) for 10 s before each measurement with a multimode microplate reader (Tecan Group Ltd., Männedorf, Switzerland). Growth curves were plotted from automatic measurement taken every 30 min. Generation time and lag phase were calculated with the software of the microplate reader. All growth kinetics studies were performed in quadruplicate.

### Tested molecule

17β-estradiol (Sigma-Aldrich, Saint-Quentin-Fallavier, France) is poorly soluble in aqueous media. We therefore first produced a stock solution in 100% ethanol. For bacterial treatment, this solution was diluted in MRS such that the final concentration of ethanol in the MRS was fixed at 0.1% v/v. Controls were performed with the same percentage of ethanol in MRS, but without estradiol. Preliminary studies were performed to check that 0.1% ethanol in MRS had no effect on *L. crispatus* CIP104459 (Suppl Fig. 3).

### Evaluation of bacterial membrane fluidity

*L. crispatus* CIP104459 was cultured in the presence or absence of 17β-estradiol over the entire course of the experiment (18 h). The bacteria were then collected by centrifugation (7500 × *g*, 10 min) and washed twice at room temperature in 10 mM MgSO_4_. The pellets were resuspended in the same solution and the OD_600 nm_ was adjusted to 0.1. We added 1 µL of 4 mM 1,6-diphenyl-1,3,5-hexatriene (DPH) in tetrahydrofuran (Sigma-Aldrich, Saint-Quentin-Fallavier, France) to each 1 mL aliquot of the bacterial suspension. Aliquots were then incubated in the dark for 30 min at 37 °C, for incorporation of the probe into the bacterial membrane. The probe was not removed, to make it possible to visualize any change in membrane fluidity. Fluorescence polarization was measured with a Spark 20 M multimode microplate reader, equipped with an active temperature regulation system (Te-Cool, Tecan Group Ltd., Männedorf, Switzerland). The excitation and emission wavelengths were set at 365 and 425 nm, respectively. Each measurement was performed in triplicate. Membrane anisotropy (r value) was calculated as described by Lakowicz^63^. Data were analyzed with SparkControl software 2.1 (Tecan Group Ltd., Männedorf, Switzerland). Fluorescence polarization is inversely related to membrane fluidity. Increases in anisotropy indicate a decrease of membrane fluidity, and vice versa. In a second series of experiments, the bacteria were cultured for 6, 12 or 24 h in normal MRS medium, collected and exposed immediately to 17β-estradiol and to the fluorescent probe. Changes in fluorescence polarization were measured over a period of 3 h, with the same equipment.

### Determination of bacterial surface polarity

The surface polarity and Lewis acid–base properties of *L. crispatus* CIP104459 with and without exposure to 17β-estradiol were studied by microbial adhesion to solvents (MATS) assays^64^. Bacteria were cultured in MRS for 18 h and harvested by centrifugation (7500 × *g*, 10 min) after 18 h of incubation. The pellets were washed twice in phosphate buffered saline (PBS) (Lonza, Thermo Fisher Scientific, Waltham, Massachusetts, USA) to remove all traces of the culture medium. Two pairs of solvents were used: chloroform/hexadecane and ethyl acetate/n‐decane were employed. For each condition, 1.2 mL of bacterial suspension at OD_400 nm_ = 0.8 was mixed for 60 s with 0.2 mL of each solvent. After incubation for 15 min and separation of the aqueous and organic phases, the OD_400 nm_ of the aqueous phase was measured. The percentage of bacteria accumulating in each organic compartment (solvent phase) was calculated with the following equation, where [AO] = OD_400 nm_ of the aqueous phase without solvent and [A] = OD_400 nm_ of the aqueous phase after exposure to the solvent:

$$\% \;{\text{solvent}}\;{\text{affinity}} = (1 - {\text{A}})/{\text{A}}0 \times 100$$

All experiments were performed at least in triplicate.

### Investigation of bacterial aggregation and morphology

We assessed the formation of aggregates by *L. crispatus* CIP104459 by the sedimentation technique described by Vandevoorde et al.^38^. Briefly, *L. crispatus* CIP104459 cultured in MRS for 18 h at 37 °C, in the presence or absence of 17β-estradiol, under anoxic conditions, without shaking, was harvested by centrifugation (7500 × *g*, 10 min, room temperature), washed twice in PBS, and resuspended in 10 mL of the same medium. After 1.5 min of agitation (vortexing), defined as T = 0 for this experiment, the change in OD_600 nm_ of the suspension was monitored over a period of 30 min with a spectrophotometer (Thermo Fisher Scientific, Waltham, Massachusetts, USA). The percentage of auto‐aggregation after 30 min was calculated as follows:

$$\% \;{\text{auto-aggregation}} = \left( {\left[ {\left( {{\text{OD}}0\;{\text{min}} - {\text{OD}}30\;{\text{min}}} \right)/{\text{OD}}0\;{\text{min}}} \right]} \right)/{\text{0D}}0\;{\text{min}} \times 100$$

where OD0 min is the initial OD_600 nm_ at T = 0 and OD30 min is the final OD_600 nm_ after 30 min.

The potential auto-aggregation and structure of *L. crispatus* CIP104459 was also studied by flow cytometry with a CytoFlex S flow cytometer (Beckman coulter Life science, Indianapolis, USA) and the CytExpert software. After 18 h of culture in the presence or absence of 17β-estradiol, the bacterial suspension was harvested by centrifugation as previously described and resuspended in PBS. Bacteria were not stained and aliquots (200 µL) were distributed into 96-well microplates (Thermo Fisher Scientific, Waltham, Massachusetts, USA). After incubation in static condition for 30 min, the samples were subjected to flow cytometry. At least 10,000 events at OD_488 ± 4 nm_ (SSC channel) and OD_525 ± 20 nm_ (FSC channel) were recorded at a flow rate of 10 µL.min^-1^ for each treatment. Data were analyzed with Cytexpert software. The percentage of the population forming aggregates corresponded to the fraction of events appearing in the Q1-UR quarter of the graph (red zone). Size is given by the FSC-A (horizontal) axis and the percentage of small bacteria appearing in the Q1-UL and Q1-LL zones. Surface granulometry is plotted on the SSC-A (vertical) axis. Increases in complexity appear in the Q1-UL and Q1-UR areas of the graph. Measurements were performed in triplicate.

The precise morphology of *L. crispatus* CIP104459 was studied by scanning electron microscopy with a TENEO VolumeScope microscope (FEI, Hillsboro, OR, USA) running at 10 kV. *L. crispatus* cultured for 18 h in the absence (control) or presence of 17β-estradiol was collected by centrifugation (7500 × *g*, 10 min). For fixation, bacterial pellets were immersed in 1 mL 2.5% glutaraldehyde in 1 M phosphate buffer, pH 7.1 for 1 h. Samples were prepared as previously described^65^. After treatment with HMDS (hexamethyldisilazane), the surface of the filter was coated with an electrically conductive 25 nm thick layer of platinum alloy coating in a sputter-coater vacuum chamber (LEICA EM ACE600 sputter coater, Wetzlar, Germany).

### Evaluation of biosurfactant production

Cultures on MRS agar in Petri dishes showed that *L. crispatus* CIP104459 grew in the presence of 17β-estradiol, spreading out and flowing over the surface of the culture medium, consistent with biosurfactant production. Petri dishes containing MRS agar were then inoculated by spreading 100 µL of control or 17β-estradiol treated bacterial suspensions over the surface to obtain a continuous bacterial lawn. As described by Meylheuc et al.^41^, we gently scraped the bacterial lawn formed after 24 h of anoxic culture at 37 °C in the presence or absence of 17β-estradiol off the plate and resuspended it in 15 mL Volvic water. The suspension was vortexed for 3 min to solubilize the biosurfactant molecules and centrifuged twice, for 30 min each, at 4 °C and 10,000 × *g* to remove all bacteria and bacterial fragments. Control solutions were generated by scraping and rinsing the surface of sterile MRS agar Petri dishes, with or without 17β-estradiol supplementation. The supernatant was collected and stored at 4 °C.

The presence of biosurfactant in the solution was first investigated by the sessile drop technique^66^, by depositing 20 µL drops of supernatant on a polystyrene surface and visualizing the angle of contact with the surface. The measurement of this contact angle can be used to calculate the surface tension between the solution and the surface. However, this value is influenced by the properties of the surface, and we therefore preferred to use another approach: the pendant drop method. The shape of the drops of supernatant was analyzed with a drop shape analyzer, a DSA30 controlled temperature tensiometer equipped with a video camera (Kruss, Hamburg, Germany). The surface tension, or interfacial tension, was calculated with the drop shape analysis software of the tensiometer, according to the Young–Laplace equation^67^. Potential variations due to the bacterial biomass collected were taken into account, by calculating the correlation between surface tension values and the OD_600 nm_ of the bacterial suspension measured immediately after the scrapping of the bacterial lawn off the Petri dishes. 17β-estradiol had no influence on the surface tension of pure water.

### Measurement of bacterial adhesion to vaginal cells

The adhesion of *L. crispatus* CIP104459 to vaginal cells was studied in vitro with the VK2/E6E7 cells line (ATCC CRL-2616). This vaginal cell line was developed from the vaginal mucosal tissue of a healthy pre-menopausal woman. It was maintained and propagated in keratinocyte serum-free medium (KSFM) (Thermo Fisher Scientific, Waltham, Massachusetts, USA), supplemented with 0.05 mg/mL bovine pituitary extract, 0.1 ng/mL human recombinant EGF and additional calcium chloride (44.1 mg/mL), as recommended by the manufacturer. Cells were transferred to fresh medium every three days after reaching confluence. For the evaluation of bacterial adhesion, VK2/E6E7 cells were exposed to *L. crispatus* CIP104459 cultured for 18 h in MRS medium with or without 17β–estradiol at an MOI of 100 bacteria/cell. Experiments were performed with antibiotic free KSFM and VK2/E6E7 cells grown to at least 80% confluence. After 1 h of bacterial interactions, the medium was gently removed to withdraw the planktonic bacteria and the surface of the plate was rinsed carefully with antibiotic free KSFM. The attached VK2/E6E7 cells were disrupted by adding of 0.1% Triton X-100 in PBS. The lysate was diluted in MRS medium and plated on MRS agar Petri dishes. The number of bacteria adhering to cells was deduced directly by counting the number of *L. crispatus* colonies after 48 h of culture at 37 °C in anoxic conditions.

### Determination of biofilm formation activity and structure

Biofilm formation by *L. crispatus* CIP104459 was initially studied with the crystal violet technique according to a procedure adapted from that of O’Toole^68^. The OD_600 nm_ of bacteria grown in MRS medium for 48 h was adjusted to 0.1 in a final volume of 1 mL, and the bacterial suspension was dispensed into sterile 24-well polystyrene plates (Falcon, Durham, USA). The plates were incubated in anoxic conditions at 37 °C without shaking for 48 h in a Whitley A85 Workstation. At the end of the incubation period, the OD_600 nm_ was measured, to determine the culture biomass. The medium was removed by aspiration and non-adherent bacteria were eliminated by washing twice in physiologic water (PW, NaCl 0.9%). Biofilms were stained by incubation with crystal violet (CV, 0.1% w/v in sterile pure 18.2 MΩ water) for 10 min at room temperature. Excess dye was removed by three washes in pure 18.2 MΩ water. The crystal violet adsorbed by the biofilm matrix and the bacteria was dissolved in absolute ethanol (1 mL/well, 10 min incubation) and the OD_595 nm_ of the solution was determined with an automated plate reader (Te-Cool, Tecan Group Ltd., Männedorf, Switzerland). Results are expressed as a percentage of the amount of biofilm formed in control conditions, as follows:

$$\% \;{\text{control}}\;{\text{biofilm}} = \left[ {\left( {{\text{AssayOD}}_{{595\;{\text{nm}}}} /{\text{AssayOD}}_{{600\;{\text{nm}}}} } \right)} \right)/\left( {{\text{ControlOD}}_{{595\;{\text{nm}}}} /{\text{Control}}\;{\text{OD}}_{{600\;{\text{nm}}}} } \right)\big)\big] \, \times 100$$

where “AssayOD_595 nm_” is the OD_595 nm_ of the test CV solution, “AssayOD_600 nm_” is the initial OD_600 nm_ of the assay culture, “ControlOD_595 nm_” is the OD_595 nm_ of the Control CV solution and “ControlOD_600 nm_” is the initial OD_600 nm_ of the control culture.

*L. crispatus* CIP104459 cultured in MRS medium was unable to adhere and form biofilms on glass, as required for optical microscopy. A more complete medium, specific for microorganisms of the vaginal microflora, “simulating genital tract secretion” (SGTS) medium, was therefore used, as described by Geshnizgani and Onderdonk^42^ (Table 1). *L. crispatus* CIP104459 was precultured in MRS and then transferred to SGTS medium for biofilm studies. 17β-estradiol, or an equivalent amount of ethanol in water, was added to the medium from the start of the biofilm formation study. We checked that 17β-estradiol had no effect on *L. crispatus* CIP104459 growth in SGTS medium in preliminary studies (Suppl. Fig. 4). For confocal microscopy, bacteria were harvested by centrifugation (7500 × *g*, 10 min) and resuspended in SGTS medium at an OD_600 nm_ of 0.1. Aliquots (1 mL) of suspension, with or without 17β-estradiol, were added to 24-well microplates with flat glass bottoms (Sensoplate, Greiner Bio-One, Germany), which were incubated for 48 h in anoxic conditions, without shaking, for biofilm formation. Wells were washed twice with PW to remove the remaining planktonic bacteria, and biofilms were stained with SYTO 9 Green Fluorescent Nucleic Acid Stain (Thermo Fisher Scientific, Waltham, Massachusetts, USA). Stained samples were examined under an LSM 710 inverted confocal laser scanning microscope (Zeiss, Marly-le-Roi, France) with the Zen 2009 software package (version 12.0.1.362). Images were reconstructed and analyzed with COMSTAT2 software. The mean thickness (μm), biomass volume (μm^3^/μm^2^) and roughness coefficient of the biofilm were calculated for a least 30 observations per treatment. All experiments were performed at least of three times.

### Bioinformatic studies

As a steroid, 17β-estradiol is an amphiphilic molecule with no reactive groups capable of directly forming covalent conjugates with dyes or tracers. Furthermore, minor chemical modifications can greatly affect its functions, due to its small size. Isotope incorporation remains the only available technique for experimental assessment of its binding to potential receptors, but this technique is subject to heavy regulatory constraints. We therefore used bioinformatics approaches to investigate potential 17β-estradiol binding site(s) in *L. crispatus*. We used BLASTp on ExPASy (https://web.expasy.org/blast) to align the draft genome of *L. crispatus* CIP104459^34^ with the annotated sequence of *L. crispatus* CO3MRSI1^43^, to improve its definition. The FASTA amino-acid sequence of the *L. crispatus* CIP104459 genome was then aligned with the sequences of the known human estrogens receptors listed in Table 2. A bacterial protein displaying high similarity and identity to human estrogens receptors was identified and subjected to molecular docking in silico with 17β-estradiol as potential ligand. We generated 3D models of *L. crispatus* proteins with RaptorX Structure^69^ and visualized them with Python Molecular Viewer V1.5.6. Predictions were based on alignments with the crystalized structure of the corresponding eukaryotic ortholog. The potential binding of 17β-estradiol to the identified sensor protein was studied with AutoDock 4.2^44^. Binding values were generated with the Lamarkian Genetic Algorithm of AutoDock 4.2. All calculations were performed with a DELL Precision T7610 computer equipped with four hard disks (4 Tb each, giving a total of 12 Tb under RAID5).

### Statistical analysis

Statistical significance was evaluated with Prism GraphPad online tool (https://www.graphpad.com/quickcalcs/ttest1/). Data were analyzed in unpaired (two sample) two-tailed *t* tests. Means and the standard error of the mean (SEM) were calculated and plotted.

### Ethics approval and consent to participate

Not applicable, as this study did not involve a clinical trial.

### Consent for publication

All the authors have read and approved the manuscript. LMSM agrees to be responsible for paying the publication fees.

## Supplementary Information

Supplementary Information 1.

Supplementary Information 2.

Supplementary Information 3.

Supplementary Information 4.

Supplementary Information 5.

## Data Availability

All experimental raw data are available, upon simple demand from the authors.
