# Supplementary material for: Effect of 17β-estradiol on a human vaginal Lactobacillus crispatus strain
Source: Sci Rep. 2021 Mar 30;11:7133. doi: 10.1038/s41598-021-86628-x (PMC8010061; doi:10.1038/s41598-021-86628-x)
Supplement: Supplementary file 1 — Supplementary Information 1. [file 41598_2021_86628_MOESM1_ESM.pptx]

## Slide 1
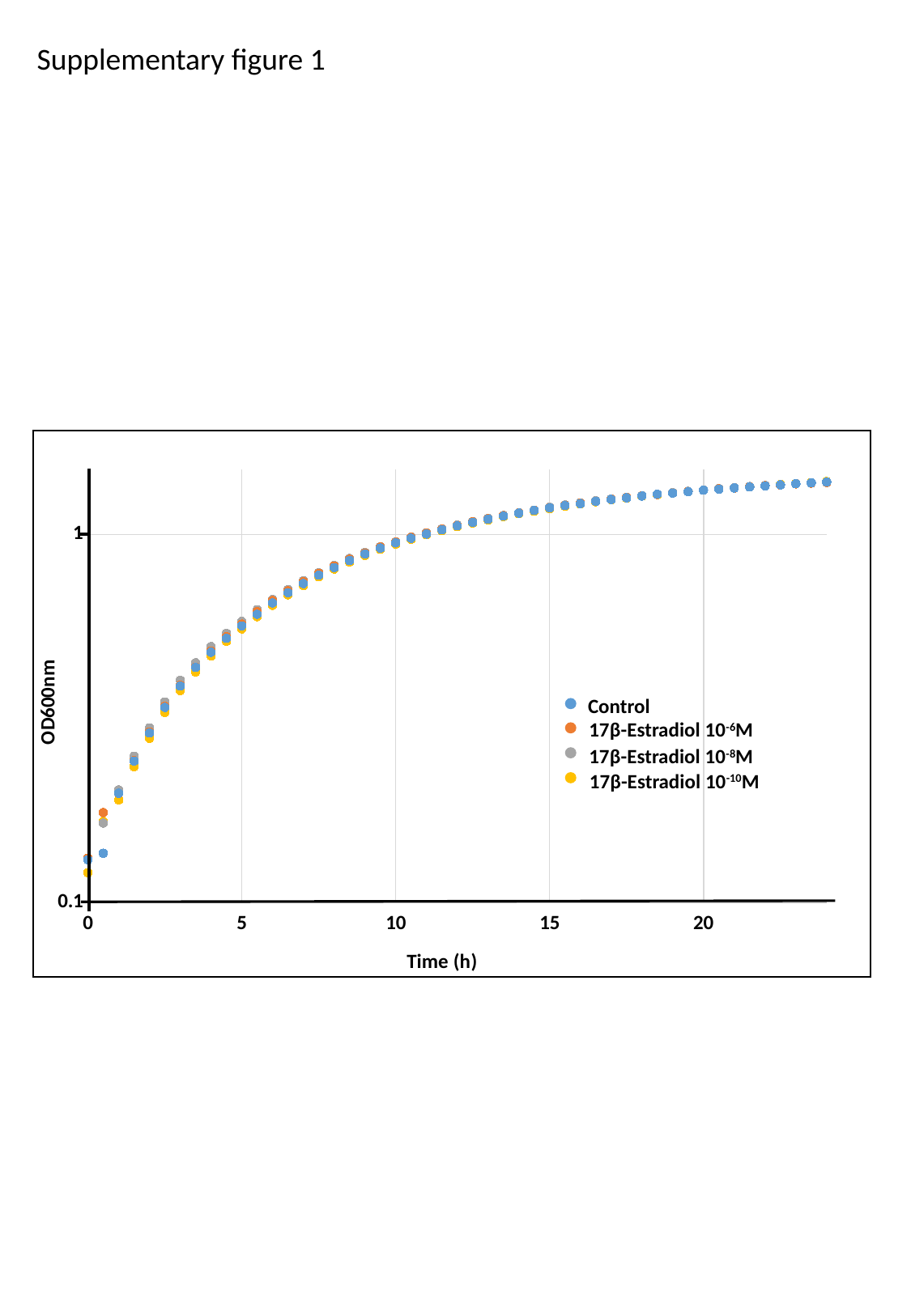

Supplementary figure 1
### Chart
| Category | Control CIP | CIP + Estradiol 10-6M | CIP + Estradiol 10-8M | CIP + Estradiol 10-10M |
|---|---|---|---|---|Control
17β-Estradiol 10-6M
17β-Estradiol 10-8M
17β-Estradiol 10-10M
