# Supplementary material for: Effect of 17β-estradiol on a human vaginal Lactobacillus crispatus strain
Source: Sci Rep. 2021 Mar 30;11:7133. doi: 10.1038/s41598-021-86628-x (PMC8010061; doi:10.1038/s41598-021-86628-x)
Supplement: Supplementary file 2 — Supplementary Information 2. [file 41598_2021_86628_MOESM2_ESM.pptx]

## Slide 1
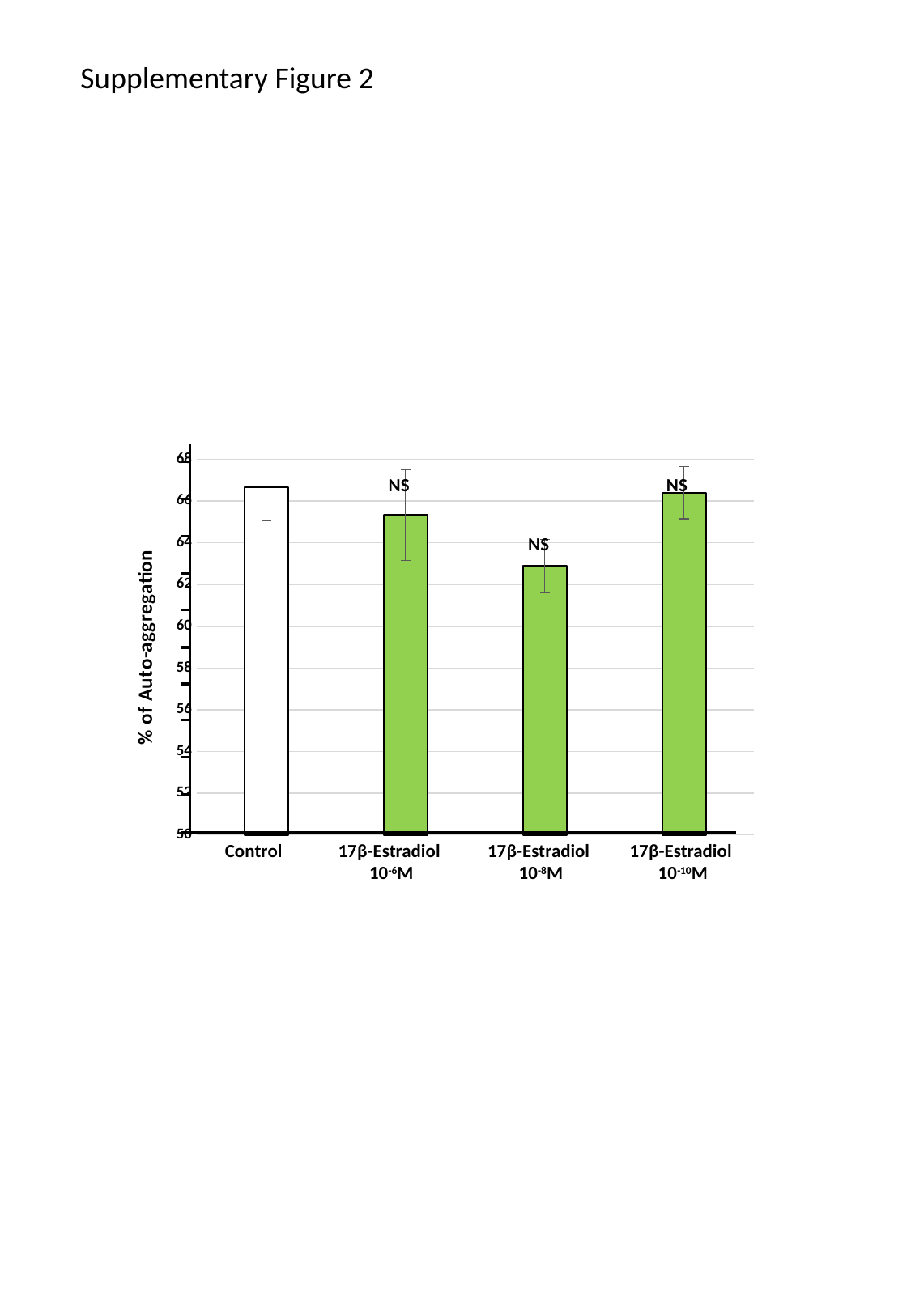

Supplementary Figure 2
### Chart
| Category | |
|---|---|
| Control | 66.64221603146996 |
| E 10-6M | 65.321772796188 |
| E10-8M | 62.88320561614522 |
| E10-10M | 66.39904489007625 |NS
NS
NS
17β-Estradiol
 10-10M
17β-Estradiol
 10-8M
Control
17β-Estradiol
10-6M
