# Supplementary figures and images for: Effect of 17β-estradiol on a human vaginal Lactobacillus crispatus strain
Source: Sci Rep. 2021 Mar 30;11:7133. doi: 10.1038/s41598-021-86628-x (PMC8010061; doi:10.1038/s41598-021-86628-x)

## Slide 1
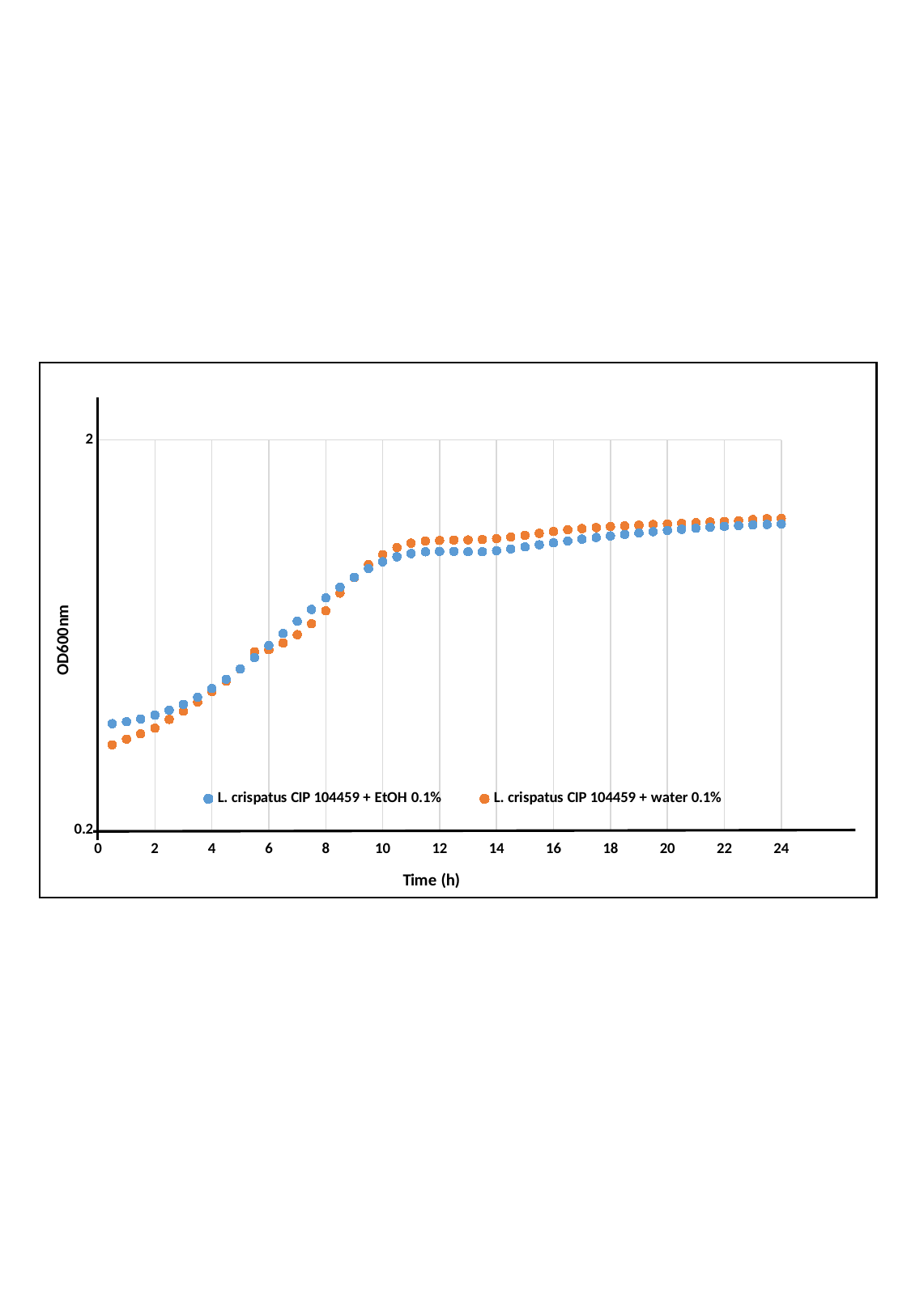

### Chart
| Category | L. crispatus CIP 104459 + EtOH 0.1% | L. crispatus CIP 104459 + water 0.1% |
|---|---|---|

Supplement: Supplementary file 3 — Supplementary Information 3. [file 41598_2021_86628_MOESM3_ESM.pptx]
