# Supplementary material for: Effect of 17β-estradiol on a human vaginal Lactobacillus crispatus strain
Source: Sci Rep. 2021 Mar 30;11:7133. doi: 10.1038/s41598-021-86628-x (PMC8010061; doi:10.1038/s41598-021-86628-x)
Supplement: Supplementary file 5 — Supplementary Information 5. [file 41598_2021_86628_MOESM5_ESM.docx]

***Supplementary data title page:***

**Effect of 17β-estradiol on a human vaginal *Lactobacillus crispatus* strain**

Maximilien CLABAUT, Amandine SUET, Pierre-Jean RACINE, Ali TAHRIOUI, Julien VERDON, Magalie BARREAU, Olivier MAILLOT, Agathe LE TIRANT, Madina KARSYBAYEVA, Coralie KREMSER, Gérard REDZINIAK, Cécile DUCLAIROIR-POC, Chantal PICHON, Sylvie CHEVALIER, Marc G.J. FEUILLOLEY

**Supplementary Figure 1.** Growth kinetics of *Lactobacillus crispatus* CIP 104459 in the presence or absence of 10^-6^, 10^-8^ and 10^-10^ M 17β-estradiol. Bacteria were cultured in MRS broth, in anoxic conditions, at 37°C, for 24h. OD_600nm_ was measured every 15 min, with an automated microplate reader. All data are the mean ± SEM of four independent experiments.

**Supplementary Figure 2**. Influence of 10^-6^, 10^-8^ and 10^-10^ M 17β-estradiol on *Lactobacillus crispatus* CIP 104459 aggregation potential. The percentage aggregation was measured 30 minutes after pellet resuspension. All data shown are the mean ± SEM of three independent experiments. (NS: not significant).

**Supplementary Figure 3.** Growth kinetics of *Lactobacillus crispatus* CIP 104459 cultured in MRS medium in the presence or absence of ethanol 0.1 %. All data are the mean ± SEM of four independent experiments.

**Supplementary Figure 4**. Growth kinetics of *Lactobacillus crispatus* CIP 104459 in simulating genital tract secretion (SGTS) medium in the presence or absence of 10^-6^, 10^-8^ and 10^-10^ M 17β-estradiol. All data are the mean ± SEM of four independent experiments.
